# Nutrient starvation leading to triglyceride accumulation activates the Entner Doudoroff pathway in *Rhodococcus jostii* RHA1

**DOI:** 10.1186/s12934-017-0651-7

**Published:** 2017-02-27

**Authors:** Antonio Juarez, Juan A. Villa, Val F. Lanza, Beatriz Lázaro, Fernando de la Cruz, Héctor M. Alvarez, Gabriel Moncalián

**Affiliations:** 1 Institut de Bioenginyeria de Catalunya, Parc Científic de Barcelona, 08028 Barcelona, Spain; 2 Departamento de Microbiología, Facultad de Biología, Universidad de Barcelona, Avda Diagonal, 643., 08028 Barcelona, Spain; 3 Departamento de Biología Molecular (Universidad de Cantabria) and Instituto de Biomedicina y Biotecnología de Cantabria IBBTEC (CSIC-UC), C/Albert Einstein 22, 39011 Santander, Spain; 4 INBIOP (Instituto de Biociencias de la Patagonia), Consejo Nacional de Investigaciones Científicas y Técnicas, Facultad de Ciencias Naturales, Universidad Nacional de la Patagonia San Juan Bosco, Ruta Provincial No 1, Km 4-Ciudad Universitaria 9000, Comodoro Rivadavia, Chubut Argentina

**Keywords:** *Rhodococcus*, Triacylglycerol, Nutrient starvation, RNA-Seq, Entner-Doudoroff pathway, CRP

## Abstract

**Background:**

*Rhodococcus jostii* RHA1 and other actinobacteria accumulate triglycerides (TAG) under nutrient starvation. This property has an important biotechnological potential in the production of sustainable oils.

**Results:**

To gain insight into the metabolic pathways involved in TAG accumulation, we analysed the transcriptome of *R jostii* RHA1 under nutrient-limiting conditions. We correlate these physiological conditions with significant changes in cell physiology. The main consequence was a global switch from catabolic to anabolic pathways. Interestingly, the Entner-Doudoroff (ED) pathway was upregulated in detriment of the glycolysis or pentose phosphate pathways. ED induction was independent of the carbon source (either gluconate or glucose). Some of the diacylglycerol acyltransferase genes involved in the last step of the Kennedy pathway were also upregulated. A common feature of the promoter region of most upregulated genes was the presence of a consensus binding sequence for the cAMP-dependent CRP regulator.

**Conclusion:**

This is the first experimental observation of an ED shift under nutrient starvation conditions. Knowledge of this switch could help in the design of metabolomic approaches to optimize carbon derivation for single cell oil production.

**Electronic supplementary material:**

The online version of this article (doi:10.1186/s12934-017-0651-7) contains supplementary material, which is available to authorized users.

## Background

Microbial triglycerides, called single cell oils (SCO), have biotechnological potential in the production of sustainable oils for their use either as biodiesel or as commodity oils. Biodiesel is produced by transesterification of triacylglycerides with short-chain alcohols (mainly methanol). Vegetable oils and animal fats such as soybean oil, rapeseed oil, palm oil or waste cooking oils are used as feedstocks for biodiesel production [1]. However, this strategy has been criticized for being a non-sustainable process since it leads to a reduction in edible oil feedstocks [2]. Production of biodiesel using SCO is considered as a promising alternative solution [3]. SCO produce high quality biodiesel esters according to currently existing standards [4, 5]. SCO are appropriate for their use as a biodiesel source since the producing microorganisms can grow using a variety of substrates, show rapid life cycles and can be easily modified by genetic engineering.

Several microorganisms, including bacteria, yeasts, molds and microalgae, can be considered as oleaginous microorganisms [6]. Regarding bacteria, the accumulation of the neutral lipids triacylglycerols (TAGs), wax esters (WEs) and polyhydroxyalkanoates (PHAs) has been reported. The main purpose of this accumulation is to store carbon and energy under growth-limiting conditions. While PHAs are synthesized in a wide variety of bacteria [7], the accumulation of triacylglycerols (TAGs) has only been described for a few bacteria belonging to the proteobacteria and actinobacteria groups (for a review see [8]). *Acinetobacter* [9] *Mycobacterium* [10], *Streptomyces* [11] or *Rhodococcus* [12] are such examples. Accumulation of TAGs is remarkably high in the actinobacteria *Rhodococcus* and *Gordonia*, which accumulate up to 80% of the cellular dry weight in the form of neutral lipids with maximal TAG production of 88.9 and 57.8 mg/l, respectively [13].

*Rhodococcus* are aerobic, non-sporulating soil bacteria, with unique enzymatic activities used for several environmental and biotechnological processes [14]. *Rhodococcus* strains are industrially used for large-scale production of acrylamide and acrylic acid as well as for the production of bioactive steroid compounds and fossil fuel biodesulfurization [15]. Moreover, *Rhodococcus* are able to degrade contaminant hydrophobic natural compounds and xenobiotics. *R. jostii* RHA1 has been shown to convert lignocellulose into different phenolic compounds [16] while it also has the potential to use this waste material for the production of valuable oils [17].

Due to its capability for degrading hydrocarbons, *R. jostii* RHA1 is one of the best studied *Rhodococcus* species in the terms of biotechnological applications [18–20]. Moreover, high TAG accumulating capability has been reported [21] and its genomic sequence is available [22].

In this article we decipher the metabolic changes associated to nutrient starvation conditions that influence TAG accumulation.

## Methods

### Bacterial strain and growth conditions

*Rhodococcus jostii* strain RHA1 was grown aerobically at 30 °C in *Streptomyces* medium, Fluka (Rich Medium, RM, 4.0 g/l glucose, 4.0 g/l Yeast extract, and 10.0 g/l Malt extract). After 48 h, 25 ml of *R. jostii* cells in RM were collected by centrifugation, washed with mineral salts medium M9 (Minimal Medium, MM, [23], 95 mM Na_2_HPO_4_, 44 mM KH_2_PO_4_, 17 mM NaCl, 0.1 mM CaCl_2_ and 2 mM MgSO_4_) containing 20% w/v sodium gluconate (MMGln) or 20% w/v glucose (MMGls) as the sole carbon sources and transfer into 25 ml of MMGln or MMGls. The concentration of ammonium chloride in MM was reduced to 10 mM to enhance lipid accumulation.

### Extraction and analysis of lipids

Pelleted cells were extracted with hexane/isopropanol (3:1 v/v). An aliquot of the whole cell extract was analyzed by thin layer chromatography (TLC) on silica gel plates (Merck) applying n-hexane/diethyl ether/acetic acid (80:20:1, v/v/v) as a solvent system. Lipid fractions were revealed using iodine vapour. Trioleine and oleic acid (Merck) were used as standards.

### RNA extraction

RNA was extracted from RM and MM-grown cells originally harvested from 3 ml of culture. Total RNA isolation involved vortexing of the pellet with 6 ml of RNA Protect (QIAGEN) followed by centrifugation. The pellet was thereafter lysed using 280 μl of lysis buffer (10% Zwittergent (Calbiochem), 15 mg/ml Lysozime (Sigma) and 20 mg/ml Proteinase K (Roche) in TE buffer). Total RNA was purified with RNeasy mini kit (QIAGEN, Valencia, CA) combined with DNase I (QIAGEN) according to the manufacturer’s instructions. The quantity and quality of RNA were assessed using a NanoDrop ND-1000 spectrophotometer (NanoDrop Technology, Rockland, DE) and Experion Automated Electrophoresis using the RNA StdSens Analysis Kit (Bio Rad).

### mRNA enrichment

Removal of 16S and 23S rRNA from total RNA was performed using MicrobExpress™ Bacterial mRNA Purification Kit (Ambion) according to the manufacturer’s protocol with the exception that no more than 5 μg total RNA was treated per enrichment reaction. Each RNA sample was divided into multiple aliquots of ≤5 μg RNA and separate enrichment reactions were performed for each sample. Enriched mRNA samples were pooled and run on the 2100 Bioanalzyer (Agilent) to confirm reduction of 16S and 23S rRNA prior to preparation of cDNA fragment libraries.

### Preparation of cDNA fragment libraries

Ambion RNA fragmentation reagents were used to generate 60–200 nucleotide RNA fragments with an input of 100 ng of mRNA. Following precipitation of fragmented RNA, first strand cDNA synthesis was performed using random N6 primers and Superscript II Reverse Transcriptase, followed by second strand cDNA synthesis using RNaseH and DNA pol I (Invitrogen, CA). Double stranded cDNA was purified using Qiaquick PCR spin columns according to the manufacturer’s protocol (Qiagen).

### RNA-Seq using the Illumina genome analyzer

The Illumina Genomic DNA Sample Prep kit (Illumina, Inc., San Diego, CA) was used according to the manufacturer’s protocol to process double-stranded cDNA for RNA-Seq. This process included end repair, A-tailing, adapter ligation, size selection, and pre-amplification. Amplified material was loaded onto independent flow cells. Sequencing was carried out by running 36 cycles on the Illumina Genome Analyzer IIx. The quality of the RNA-Seq reads was analyzed by assessing the relationship between the quality score and error probability. These analyses were performed on Illumina RNA-Seq quality scores that were converted to phred format (http://www.phrap.com/phred/).

### Computational methods

To filter genes with low signal/noise ratio we built 3 subsets of each condition taking randomly 70% of the total sequenced reads for each subset. The alignment was performed by Bowtie [24] against the *R. jostii* RHA1 reference genomes of the chromosome and three endogenous plasmids (Genome Reviews CP000431-4_GR). Gene expression was determined by Samtools [25], Artemis [26] and home-made perl scripts. We represent gene expression as reads per kilobase (RPK) and the data was normalized by quantiles according to [27]. Statistical analysis was performed by DESeq package [28] and R software.

### Quantitative real-time RT-PCR (qRT-PCR)

cDNA was generated from 1.5 µg of total RNA using the iScript kit (BioRad) according to manufacturer’s instructions. 1 µl of the cDNA template was then used in quantitative real-time PCR reactions using iQ SUYBRE Green Supermix (BioRad) and a iCycler iQ5(BioRad). Primers were designed using Primer3 (http://primer3.sourceforge.net). The cycle of threshold (Ct) was determined for each reaction using the iQ5 Optical System Software 2.0 (BioRad). All qRT-PCR reactions were done in triplicate.

### KDPG aldolase activity assay

KDPG aldolase activity was quantified by a lactate dehydrogenase (LDH) coupled assay where the production of pyruvate is related to the NADH consumption, as described in [29]. 2 ml of *R. jostii RHA1* RM or MMGls cultures were harvested and resuspended in 1 ml of buffer TrisHCl 100 mM pH 7.5, NaCl 300 mM, EDTA 1 mM, DTT 1 mM and PMSF 1 mM. The cells were lysed using 0.2 mm silica beads and a Fast Prep-24 system (MP Biomedicals) for 3 cycles of 60 s and centrifuged at 100,000*g* for 25 min at 4 °C. 150 μl aliquots of the resulting RM or MMGls total extracts were then treated with 1 μl of LDH (5 U/μL), 0.70 μl of NADH (50 mM) and 1 μl of KDPG (50 mM). Decrease in NADH absorbance at 340 nm was measured in quartz microcuvettes (150 μl) in a UV-1603 spectrophotometer (Shimadzu) for 5 min. Total protein concentration was determined by Bradford assays using BSA as standard. KDGP activity was calculated as moles of NADH consumed per mg of total protein per second (mol/s/mg).

## Results and discussion

### Culture conditions for *R. jostii* RHA1, TAGs accumulation and RNA-Seq analysis

**Fig. 1:**
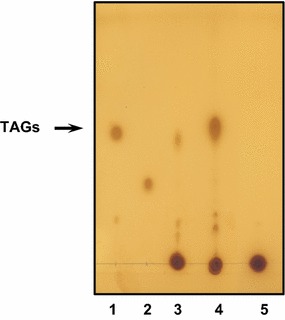
TLC analysis of the crude organic extracts obtained from the *R. jostii* RHA1 cultures used for RNA-Seq. Cells were grown in RM or MMGln media prepared as described in "Methods" section. Lipids were extracted and separated by TLC on silica gel plates, solvent extract: hexane/2-isopropane acid (3:1 v/v). *Lane 1* control trioleine;* 2* control oleic acid;* 3* Cells grown 4 h in MMGln;* 4* Cells grown 8 h in MMGln;* 5* Cells grown 24 h in RM. *R jostii* isolated TAGs are shown by a *black arrow*

*R. jostii* RHA1 is able to transform a diverse range of organic substrates into large quantities of TAGs [21]. The best conditions for TAG accumulation in *R. opacus* occur when gluconate is used as carbon source in a nitrogen-limited medium [30]. We have checked TAG accumulation over time in *R. jostii* RHA1 cells transferred to M9 medium with 10 mM ammonium chloride and sodium gluconate (20% w/v) as carbon source (MMGln medium, Fig. 1). While TAG accumulation was already detected upon 4 h in MMGln (Fig. 1), no TAG accumulation was observed at any time in a complex rich-nutrient medium (RM). TAGs were also accumulated in an M9 medium with 20 mM ammonium chloride (MMN) and even when MMN was enriched with 0.2% casamino acids (data not shown). Thus, for comparative analysis of the *R. jostii* transcriptome under conditions that lead or do not lead to TAG accumulation, RNA-Seq analyses were performed on two RNA samples collected from *R.jostii* RHA1 strain incubated either 24 h in RM medium (exponential phase) or 4 h in MMGln after 48 h in RM medium. cDNA was generated from mRNA-enriched total RNA preparations from each strain and sequenced using the Illumina Genome Analyzer IIx as described in Methods, to yield a total number of 9,611,145 reads for MMGln and 14,330,620 reads for RM (Table 1).

**Table 1:** Summary of the *R. jostii* cDNA samples sequenced using the Illumina genome analyzer

Sequenced sample	Total mapped reads	Total mapped bps (×10^6^)	Mapped mRNA reads	Mapped mRNA bp (×10^6^)	mRNA reads (% of all mapped reads)
MMGln	9,611,145	336.39	2,751,223	96.292	28.6
RM	14,330,620	501.57	1,554,502	54.408	10.8

**Fig. 2:**
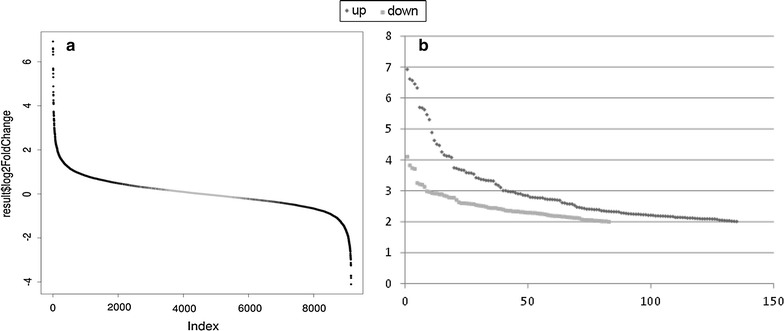
Differential expression of the 9145 genes of *R. jostii* RHA1. **a** Global differential expression. *Black spots* represent a p value lower than 0.001. **b** Upregulation (*black dots*) or downregulation (*grey dots*) levels in MMGln

For comparative analysis of the *R. jostii* transcriptome under conditions that lead or do not lead to TAG accumulation, reads per kilobase (RPK) were calculated for each of the 9145 annotated *R jostii* genes [22] and normalized for each condition as described in “Methods” section (Additional file 1: Table S1). After data processing, we observed 701 upregulated genes (twofold or greater, MMGln vs RM) and 538 downregulated genes (twofold or greater, MMGln vs RM) (Table 2; Fig. 2a). Whereas the percentage of chromosomal upregulated and downregulated genes was similar (6.3 vs 6.8%), the percentage of plasmid upregulated genes was much higher than the percentage of downregulated genes (13.3 vs. 2.0% in pRHL1, 11.7 vs. 4.4% in pRHL2 and 11.4 vs. 0.9% in pRHL3) (Table 2). Predominant gene upregulation is a common feature of different bacterial stress conditions where a quick response to environmental changes is needed [31]. It is also apparent that, for the whole genome, genes showing high induction predominate over genes showing high repression (Fig. 2b). 42 genes showed eightfold or higher upregulation, while only 8 genes showed eightfold or higher downregulation (Additional file 1: Table S1).

**Table 2:** Distribution of the upregulated and downregulated genes in the chromosome and plasmids of *R. jostii* RHA1

	pRHL3	pRHL2	pRLH1	Chrom	Total
Up	38	53	153	457	701
Normal	293	381	970	6262	7906
Down	3	20	23	492	538
All	334	454	1146	7211	9145

	%pRHL3	%pRHL2	%pRHL1	%Chrom	Total
Up	11.38	11.67	13.35	6.34	7.66
Normal	87.72	83.92	84.64	86.84	86.45
Down	0.90	4.40	2.00	6.82	5.88
All	100	100	100	100	100

### Comparative analysis of *R. jostii* RHA1 transcriptome under nutrient-rich and nutrient-limiting (TAG accumulating) conditions

**Fig. 3:**
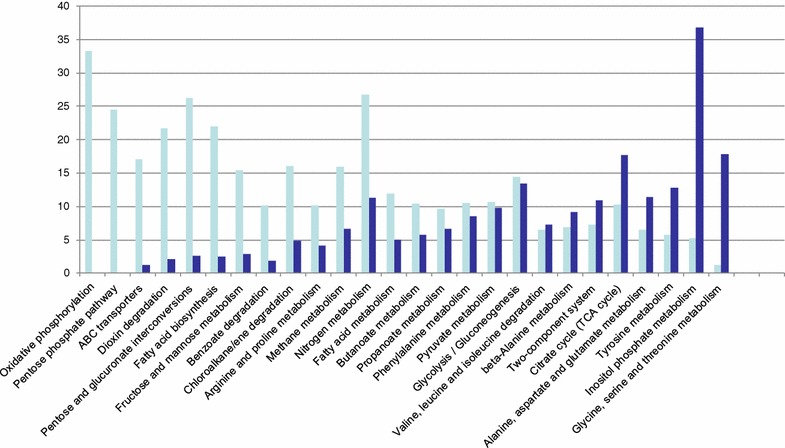
Number of up- and downregulated MMGln *R. jostii* genes in the corresponding KEGG functional pathways. The *bars* represent the number of genes with upregulation of twofold or greater (*cyan bars*) or a downregulation of twofold or greater (*blue bars*)

For an overview of the metabolic changes that occurred after nutrient deprivation maintaining the carbon source excess, we identified the KEGG pathways [32] corresponding to the up- or downregulated genes. For some functional categories (i.e., oxidative phosphorylation, pentose phosphate, ABC transporters, fatty acid metabolism), upregulated genes predominate (Fig. 3). In contrast, for other categories (i.e., amino acids metabolism and inositol phosphate metabolism), downregulated genes predominate. To better understand the global effects of nutrient deprivation, we looked at specific pathways rather than to functional categories. Downregulation is the rule in several metabolic activities, both catabolic and biosynthetic, as well as in the turnover of macromolecules. Key assimilatory pathways were repressed (Phosphate and sulphate assimilation, synthesis of glutamine synthetase, synthesis of C1-carriers). DNA duplication machinery and several biosynthetic pathways (i.e., pyrimidine, peptidoglycan) were also repressed. With respect to the catabolic pathways, repression occurred in: (i) degradation of several alternative carbon sources and (ii) sugar transport via phosphotransferase system (PTS). Turnover by RNA degradation was also repressed. These downregulated pathways can be interpreted as a result of cells stopping metabolic activities that lead to cell proliferation as a consequence of nutrient starvation.

Other alterations in gene expression can be directly correlated to specific starvation conditions: excess of the carbon source or depletion of the nitrogen source. Hence, significant alterations of metabolic pathways are related to nitrogen starvation: (i) amino acid catabolism is repressed and (ii) reactions that might render free ammonia from organic compounds are induced (i.e., formamidase and ethanolamine ammonia lyase). Finally, a set of metabolic activities are induced as a consequence of the fact that nutrient-starved cells can still incorporate the carbon source leading, for instance, to the synthesis of TAGs. In fact, induction of glycerol-3P-acyltransferase, fatty acid synthesis, acyl-carrier protein and biotin biosynthetic enzymes was observed. The transcriptome analysis of *R. opacus* PD630 under TAG accumulating conditions has been recently reported [33]. 3 h after cells were transferred to a minimal medium (MSM3) similar to our MMGln medium, 21.15% of the genes were upregulated >2-fold and 9.36% downregulated >2-fold. Globally, genes related to biogenesis were upregulated while genes involved in energy production or carbohydrate metabolism were downregulated. 4273 *R. jostii* RHA1 homologous genes have been found in *R. opacus* PD630 chromosome. Most of the upregulated genes in *R. jostii* MMGln are also upregulated in *R. opacus* MSM3 (Additional file 1: Table S3), thus confirming the metabolic shift observed for *R. jostii* under TAG accumulating conditions.

### Genes of the Entner-Doudoroff (ED) pathway are highly upregulated

**Fig. 4:**
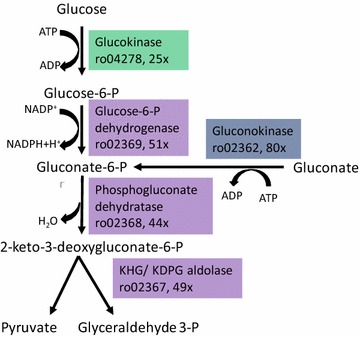
Differential expression of the genes involved in the Entner-Doudoroff pathway analysed by RNA-Seq. The *R. jostii* RHA1 gene numeration is shown together with the times the gene is upregulated in MMGln conditions

Switching metabolism to the synthesis of TAGs not only requires the upregulation of enzymes specifically involved in the corresponding biosynthetic pathways, but also the upregulation of the corresponding pathways that generate the appropriate building blocks, ATP and reducing power [34]. One of the main functional categories presenting upregulated genes that were activated when *R. jostii* cells were grown in MMGln was the pentose phosphate pathway (Fig. 3). However, a detailed analysis of the specific genes of this functional category that are upregulated showed them to belong to the ED catabolic pathway. The ED pathway is, in addition to the Embden-Meyerhof-Parnas (EMP) and pentose phosphate pathways, one of three pathways that process 6-carbon sugars [35, 36]. The first step in the ED pathway is the formation of gluconate-6-phosphate by oxidation of glucose-6-phosphate or phosphorylation of gluconate. Then, the 6-phosphogluconate dehydratase catalyzes the dehydration of 6-phosphogluconate to produce KDPG. Finally, the cleavage of KDPG catalysed by the KDPG aldolase yields pyruvate and glyceraldehyde-3-phosphate. Electrons drawn in reactions catalysed by the glucose-6P-dehydrogenase are transferred to NADP^+^. According to the RNA-Seq transcriptomic analysis, every gene coding for the different enzymes of the ED pathway was highly upregulated in the MMGln conditions (Fig. 4; Table 3).

**Table 3:** A subset of the *R. jostii* RHA1 most upregulated genes in the MMGln nutrient-deprived medium

Gene ID	RPK RM	RPK MMGln	FoldChange	Protein name
RHA1_ro02363	1190	144,105	121	Gluconate permease family protein
RHA1_ro04139	1035	101,265	98	Metabolite transporter, MFS superfamily
RHA1_ro06058	610	57,816	95	Possible ATP-dependent protease
RHA1_ro06057	1465	128,281	88	Probable 1,3-propanediol dehydrogenase
RHA1_ro02362	2544	203,714	80	Probable gluconokinase
RHA1_ro02369	2036	105,557	52	Glucose-6-phosphate 1-dehydrogenase
RHA1_ro04138	1740	89,076	51	Possible hydratase
RHA1_ro02367	2703	133,001	49	KHG/KDPG aldolase
RHA1_ro02368	2874	126,594	44	Phosphogluconate dehydratase
RHA1_ro06059	569	22,385	39	Hypothetical protein
RHA1_ro04137	702	20,726	30	Reductase
RHA1_ro04278	2020	49,893	25	Glucokinase
RHA1_ro04140	3796	86,218	23	Probable phosphoglycerate dehydrogenase
RHA1_ro02361	2216	49,009	22	Probable lipase
RHA1_ro03288	1117	21,311	19	Probable glutamate dehydrogenase (NAD(P) +)
RHA1_ro04279	2964	52,890	18	Possible transcriptional regulator, WhiB family
RHA1_ro03287	1634	28,443	17	Hypothetical protein
RHA1_ro06083	1756	30,473	17	Probable ethanolamine permease, APC superfamily
RHA1_ro01051	1414	23,886	17	Hypothetical protein

Consistently, genes involved in ED pathway were also found amongst the genes upregulated in the TAG accumulating medium in *R. opacus* PD630 (Additional file 1: Table S3).

For RNA-Seq transcriptomic analysis, we used gluconate as a carbon source in MMGln because gluconate led to the highest level of TAG accumulation in *R. opacus* [30]. Therefore, induction of the ED pathway could be the consequence of the use of gluconate as the sole carbon source and not of a general mechanism for TAG accumulation under nutrient-deprived conditions. To solve this question, we tested whether the presence of glucose in MMGls also induces TAG accumulation and the ED pathway in *R. jostii*. TAG accumulation in MM containing either glucose or gluconate as carbon source was evaluated by fluorescence measurements using red nile and the Victor-3 fluorometer system (Perkin Elmer). We observed that glucose was also able to induce TAG accumulation in *R jostii*, but to a lower extent than gluconate (data not shown). Two likely hypotheses to explain this are: (i) only gluconate is able to induce the ED pathway and glucose is metabolized to TAG by the EM pathway, or (ii) glucose is also metabolized by the ED pathway but with a slightly lower yield, because glucose has to be transformed first to gluconate.

To check if glucose was also able to activate the ED pathway under nutrient-limiting conditions, we used RT-qPCR to measure the expression of the most upregulated genes involved in the ED pathway. The expression of these genes was compared in RM and in MM with gluconate or glucose as carbon source. As shown in Table 4, the three selected genes (ro2369: glucose-6-phosphate 1-dehydrogenase, ro02367: KHG/KDPG aldolase, and ro02362: gluconokinase) were again highly upregulated when gluconate was used as carbon source in the nutrient-limited medium. Interestingly, similar upregulation was observed when the MM contained glucose instead of gluconate. Thus, the ED is also activated with glucose as carbon source supporting that the activation is due to the metabolic stress and not due to the use of gluconate as carbon source. We have selected the gene ro00588 (cold shock protein) as control or housekeeping gene. Expression of this gene led to a 1.008 fold change (MMGln vs RM) in RNA-Seq and it was also almost unaffected in any of the three used media in the RT-qPCR experiment (Table 4).

**Table 4:** qRT-PCR evaluation of the ED pathway gene expression in MM medium containing glucose or gluconate as sole carbon source

Gene	Annotation	Carbon source	Ct^a^	ΔCt^b^	FoldChange
ro02362	Probable gluconokinase	RM	30.254	–	
		MMGln	25.539	5.135	35.14
		MMGls	26.316	4.335	20.18
ro02367	KHG/KDPG aldolase	RM	24.194	–	
		MMGln	20.708	3.378	10.39
		MMGls	21.324	2.762	6.78
ro02369	Glucose-6-P 1-dehydrogenase	RM	24.084	–	
		MMGln	20.857	3.129	8.75
		MMGls	22.018	2.478	5.57
ro00588	Cold shock protein	RM	21.195	–	
		MMGln	21.086	−0.109	0.92
		MMGls	21.596	0.401	1.32

^a^ Ct is the cycle threshold or number of cycles requires for the fluorescence signal to cross the threshold. The Cts shown are the mean of three experiments

^b^ ΔCt = Ct (MM) − Ct (RM)

We have also analysed the enzymatic activity of the KHG/KDPG aldolase in crude extracts of *R. jostii RHA1* grown on MMGls or RM as described in Methods. In accordance with the transcriptomic results, KDPG aldolase activity (Additional file 2: Figure S1) was 8.75 times higher in MMGls (3.5 nmol/s/mg) than in RM (0.4 nmol/s/mg).

Catabolism of the carbon source (either glucose or gluconate) by the ED pathway renders two moles of pyruvate per mole of carbon source. One mole of ATP is generated also. However, generation of reduced coenzymes depends on the carbon source. Whereas catabolism of 1 mol of glucose by the ED pathway generates 1 mol NADPH and 1 mol NADH, catabolism of gluconate generates only 1 mol NADH (see below).

### Energy and redox metabolism in *R. jostii* RHA1 cells grown in MMGln

More than 30 genes that code for proteins of the oxidative phosphorylation process are upregulated and none of these genes is downregulated (Fig. 3). More specifically, the upregulated genes mainly code for subunits of the complex I or NADH dehydrogenase, while the genes of the F1-ATPase remain unchanged. Hence, respiratory activity may provide part of the ATP required for TAG biosynthesis.

The highest transcriptional repression was observed for the ro03923 gene coding for a NADPH dehydrogenase (Table 5). Oxidation of glucose to pyruvate by the EMP has a net yield of 2 ATP and 2 NADH per molecule of glucose. In contrast, if the ED pathway is used, the net yield is 1 ATP, 1 NADH and 1 NADPH per molecule of glucose. It should be pointed out here that if, instead of glucose, gluconate is oxidized by the ED pathway, the net yield should be 1 ATP and 1 NADH per molecule of gluconate (see Fig. 4). According to [37], the synthesis of fatty acids requires stoichiometric amounts of ATP and acetyl-CoA, NADPH and NADH for each C2 addition. Considering that catabolism of gluconate to pyruvate by the ED pathway renders NADH and not NADPH, there is a requirement for this latter reduced coenzyme for TAG biosynthesis. This may explain the downregulation of the NADPH dehydrogenase (ro03923, 0.06x).

**Table 5:** A subset of the *R. jostii* RHA1 most downregulated genes in the MMGln nutrient-deprived medium

Gene ID	RPK RM	RPK MMGln	FoldChange	Protein name
RHA1_ro04379	10,415	1519	0.146	Transcriptional regulator, GntR family
RHA1_ro04433	18,405	2666	0.145	Hypothetical protein
RHA1_ro03412	1295	183	0.142	Hypothetical protein
RHA1_ro02813	65,807	9173	0.139	Probable NADP dependent oxidoreductase
RHA1_ro03320	27,980	3784	0.135	Pyruvate dehydrogenase E1 component beta subunit
RHA1_ro04380	9371	1267	0.135	Probable multidrug resistance transporter, MFS superfamily
RHA1_ro01994	57,602	7661	0.133	Probable succinate-semialdehyde dehydrogenase (NAD(P) +)
RHA1_ro05024	76,619	10,173	0.133	Reductase
RHA1_ro03319	29,880	3927	0.131	Dihydrolipoyllysine-residue acetyltransferase, E2 component of pyruvate dehydrogenase complex
RHA1_ro03811	58,271	7458	0.128	Probable carboxylesterase
RHA1_ro03321	39,387	4982	0.126	Pyruvate dehydrogenase E1 component alpha subunit
RHA1_ro06364	18,591	2126	0.114	Probable cyanate transporter, MFS superfamily
RHA1_ro03916	27,486	2996	0.109	Hypothetical protein
RHA1_ro01380	88,898	9590	0.108	Hypothetical protein
RHA1_ro03318	48,005	5041	0.105	Dihydrolipoyl dehydrogenanse
RHA1_ro03207	21,864	1671	0.076	Hypothetical protein
RHA1_ro03206	48,313	3638	0.075	Dehydrogenase
RHA1_ro03208	38,493	2729	0.071	Polysaccharide deacetylase
RHA1_ro03923	62,548	3639	0.058	NADPH dehydrogenase

Different metabolic pathways lead to acetyl-CoA generation from pyruvate. Pyruvate dehydrogenase, partially repressed, may account for the conversion of a fraction of the total pyruvate available to acetyl-CoA. Induction of other enzymes, such as acetyl-CoA synthase (8 homologs in RHA1 like ro04332 and ro11190, 6.9× and 5.9× upregulated, respectively) (Additional file 1: Table S1), that can generate acetyl-CoA from acetate without a requirement for NAD^+^ suggests that a fraction of the available pyruvate could be converted to acetyl-CoA by enzymes that do not generate NADH.

### Induction of the Kennedy pathway for TAG accumulation

**Fig. 5:**
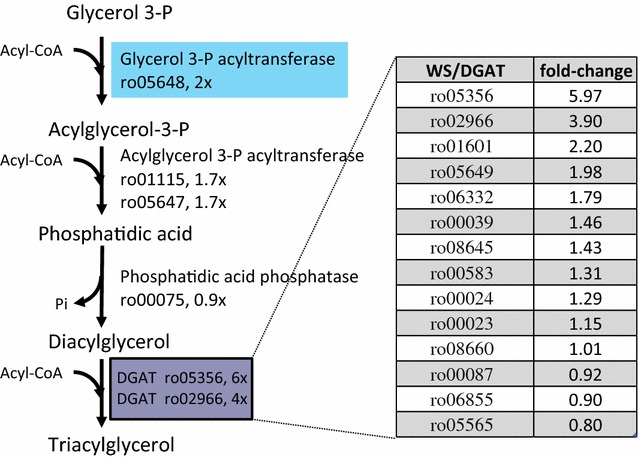
Differential expression of the genes involved in the Kennedy pathway for TAG synthesis analysed by RNA-Seq. The expression of the 14 putative *R. jostii* WS/DGAT genes is shown

The glyceraldehyde-3-phosphate generated by the ED enzyme KDPG aldolase could be used for pyruvate formation, but also for conversion to dihydroxyacetone-phosphate by a reaction catalyzed by the triose-phosphate isomerase enzyme (TpiA). Then, the dihydroxyacetone-phosphate intermediate may be converted into glycerol-3-phosphate by a NAD(P)-dependent glycerol-3-phosphate dehydrogenase enzyme (GpsA). Glycerol-3-phosphate is later sequentially acylated, after removing the phosphate group, to form TAG (Kennedy pathway). Interestingly, the genes *tpiA* (ro07179, 1.76×) and *gpsA* (ro06505, 1.78×) were both upregulated to some extent by cells during cultivation in nutrient starvation conditions. Moreover, genes involved in the de novo fatty acid biosynthesis were also upregulated. An acetyl-CoA carboxylase enzyme (ACC) coded by ro04222 (2.36×) was significantly induced in starved cells. ACC catalyzes the formation of malonyl-CoA molecules, which are used for fatty acid biosynthesis by the enzymatic complex known as fatty acid synthase I (FAS-I). FAS-I, a unique, large protein with different catalytic activities, is responsible for fatty acid biosynthesis in rhodococci, which are used for phospholipids and TAG synthesis. FAS-I coded by ro01426 (2.81×) was highly upregulated in cells under nutrient starvation conditions. Although the genes coding for several enzymes of the Kennedy pathway were not significantly upregulated in MMGln, some of the diacylglycerol acyltransferase genes were indeed upregulated (Fig. 5). The acyltransferase enzymes involved in the upper reactions of the Kennedy pathway were slightly upregulated in MMGln, such as ro05648 (GPAT) 1.99×, ro01115 (AGPAT) 1.67×, and ro05647 (AGPAT) 1.70× (Fig. 5 and Additional file 1: Table S1). Wax ester synthase/acyl coenzyme A:diacylglycerol acyltransferases (WS/DGATs) are key bacterial enzymes that catalyze the final step of TAG biosynthesis (acylation of DAG intermediates). Fourteen WS/DGAT genes were identified in *R. jostii* [21]. The WS/DGAT genes ro05356 (Atf8) and ro02966 (Atf7) were upregulated almost sixfold and fourfold, respectively. Indeed, *atf8* transcripts were also the most abundant WS/DGAT transcripts during RHA1 grow on benzoate under nitrogen-limiting conditions, being this enzyme determinant for TAG accumulation [16]. Moreover, the genes ro01601 (Atf6) and ro05649 (Atf9) were expressed 2 times more in MMGln than in RM. These four WS/DGAT enzymes are expected to be specifically involved in the TAG synthesis. Finally, ro02104 (*tadA*), another gene described to be involved in TAG accumulation, was upregulated 3.7 times in MMGln (Additional file 1: Table S1). TadA is a predicted apolipoprotein associated with lipid droplets in *R. jostii RHA1* [38] and *R. opacus PD630* [33]. *TadA* mutant was described to accumulate 30–40% less TAG than the parental *R. opacus PD630* strain [39]. This protein may mediate lipid body formation in TAG-accumulating rhodococcal cells with a similar structural role than apolipoproteins in eukaryotes [39].

### Putative CRP binding sites are present in the highly expressed genes

Alternative sigma factors such as sigma54 are widely used in bacteria as a quick response to cope with environmental changes such as nutrient deprivation. To find if these alternative factors are being used for the upregulation of the *R. jostii* genes in MMGln, the program BPROM (http://www.softberry.com/) for the recognition of sigma70 promoters was used with the 150 bp immediately upstream from each ORF start. A putative sigma70 binding site was found in most upregulated genes. Hence, regulatory element(s) alternative to sigma70 subunit must be responsible for the transcriptional activation of the *R. jostii* genes in MMGln. These element(s) should target conserved binding sites in some of the altered genes.

**Fig. 6:**
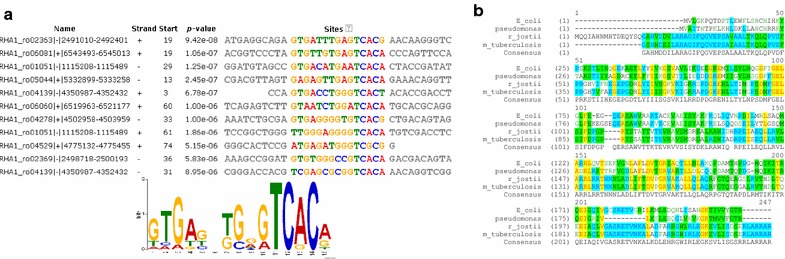
**a** Conserved sequences found by using the meme program within the 11 most upregulated *R. jostii* promoters in MMGln. The consensus sequence is also shown. **b** Alignment of the *R. jostii* putative CRP sequence (YP_704269) with the CRP sequences of *E. coli* (PDB 1O3Q), *P. Aeruginosa* (PDB 2OZ6) and *M. tuberculosis* (PDB 3D0S)

The identification and localization of conserved sequences within the upstream regions of the upregulated genes was performed by the MEME Suite [40]. The consensus sequence 5′-GTGANNTGNGTCAC-3′ was found in almost every promoter region of the 40 highest upregulated genes, as shown in Additional file 1: Table S2 and Fig. 6a. This conserved sequence is identical to the cAMP Receptor Protein (CRP) consensus binding site found either in *E. coli* (5′-tGTGANNNNNNTCACa-3′, [41]) or *Pseudomonas aeruginosa* (5′-ANWWTGNGAWNYAGWTCACAT-3′ [42]. Moreover, the protein coded by ro04321 is 90% identical (Fig. 6b) to the corresponding CRP protein in *Mycobacterium tuberculosis* [43]. Structural modelling by Phyre 2 [44] of the putative *R. jostii* CRP correctly predicts a CRP fold with 223 residues (92%) modelled at >90% accuracy.

Bacterial CRPs are transcription factors that respond to cAMP by binding at target promoters when cAMP concentration increases. 254 CRP-binding sites have been found in *E. coli*, regulating at least 378 promoters [41]. In *R. jostii*, 371 putative CRP binding sites have been found (Additional file 1: Table S2). Thus, there is a CRP binding site per, approximately, each 25 genes. However, the density increases significantly up to 1 site per 4 genes in the genes that we identified as highly upregulated (eightfold or greater) when *Rhodococcus* cells grow in MMGln. Specifically, in all the promoters controlling genes involved in the ED pathway there is at least one CRP binding site. Most of these promoters are divergent promoters and both of the controlled operons are upregulated. Moreover, CRP binding sites have also been found in the promoter regions of the two main upregulated WS/DGAT genes (ro05356 and ro02966), but not in the promoter regions of the other WS/DGAT genes. Strikingly, the promoter regions of the most upregulated operons in *R. opacus* PD630 also contain a CRP putative binding sequence (Additional file 1: Table S3).

In *E. coli*, gluconate was shown to lower both CRP and cAMP to nearly the same extent as glucose [45]. Hence, it is likely that in *R. jostii*, the predicted cAMP increase, rather than being related to the carbon source, is related to the stress generated by depletion of nutrients.

We also searched for the presence of a CRP binding site in the upstream regulatory region of the orthologs of the 40 *Rhodococcus* genes in other microorganisms using the MEME Suite (Additional file 1: Table S4). According to the results, it seems that the CRP mediated activation of the ED pathway is only conserved in *R. opacus*, also an oleogenic rhodococci. CRP binding sites were also found in the promoter regions of a few genes in the other two *Rhodococcus* genomes analyzed (*R. equi* and *R. erythropolis*). However, no consensus CRP binding sequence was found in the promoter regions of the orthologous genes in *Escherichia coli* or *Pseudomonas putida*. We have also searched without success for CRP binding sites in similar operons of non-oleaginous organisms containing WS/DGAT enzymes, such as *Mycobacterium tuberculosis*, *Acinetobacter baumanii* or *Marinobacter aquaolei*. Thus, it seems the upregulation of these *R. jostii* genes by CRP is related to the TAG accumulation.

## Conclusions

Different microorganisms are able to accumulate TAGs or other neutral lipids to serve as carbon and energy sources during starvation. One of these microorganisms is *R. jostii* strain RHA1. Transcriptomic analysis of *R. jostii* RHA1 under conditions that lead or do not lead to TAG accumulation allowed us to identify the metabolic pathways that are relevant for oxidation of the carbon source, biosynthesis and TAG accumulation under nutrient-deprivation.

Two interesting results arose from our work. First, under nutrient-deprivation, *Rhodococcus* metabolizes carbohydrates such as glucose or gluconate by the Entner-Doudoroff pathway. Up- or downregulation of other key enzymes (i.e., pyruvate dehydrogenase, acetyl CoA synthetase, NADH oxidase), provides the ATP, reducing equivalents and building blocks for TAG synthesis. Second, the metabolic shift is likely driven by an increase in cAMP concentration that activates the expression of several operons via CRP.

Both observations could help in engineering metabolic modifications to improve TAG yield for biotechnological applications.

## Additional files

**Additional file 1: Table S1.** RPK values of all genes. **Table S2.** CRP binding sequences in *R. jostii*. **Table S3.** *R. jostii* RHA1comparison to *R. opacus* PD630. **Table S4.** CRP binding sequences in rhodoccoci.

**Additional file 2: Figure S1.** (A) Kinetic determination of KDPG aldolase activity in MMGls and RM. (B). KDPG aldolase activity calculated from the kinetic curves. Error bars show the standard deviation from three independent experiments.
