# Supplementary figures and images for: Nutrient starvation leading to triglyceride accumulation activates the Entner Doudoroff pathway in Rhodococcus jostii RHA1
Source: Microb Cell Fact. 2017 Feb 27;16:35. doi: 10.1186/s12934-017-0651-7 (PMC5327559; doi:10.1186/s12934-017-0651-7)

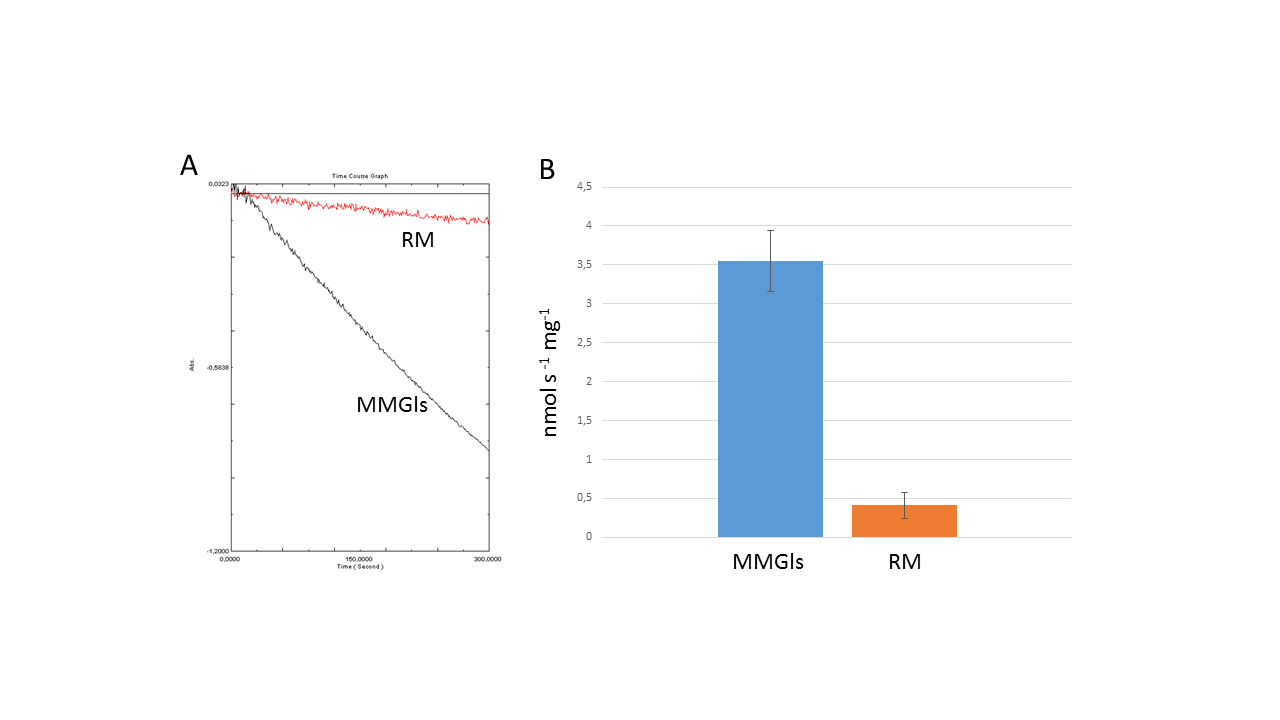

Supplement: Supplementary file 2 — Additional file 2: Figure S1. (A) Kinetic determination of KDPG aldolase activity in MMGls and RM. (B). KDPG aldolase activity calculated from the kinetic curves. Error bars show the standard deviation from three independent experiments. [file 12934_2017_651_MOESM2_ESM.tif]
